# Longitudinal evaluation of fatigue in adult patients with spinal muscular atrophy and the impact of disease-modifying drugs

**DOI:** 10.1055/s-0045-1814377

**Published:** 2025-12-22

**Authors:** Felipe Franco da Graça, Cristina Iwabe, Marcondes Cavalcante França Jr

**Affiliations:** 1 Universidade Estadual de Campinas, Faculdade de Ciências Médicas, Departamento de Neurologia, Campinas SP, Brazil.

**Keywords:** Fatigue, Muscular Atrophy, Spinal, Drug Therapy, Quality of Life, Treatment Outcome

## Abstract

**Background:**

Fatigue is a frequent and under-recognized symptom in adults with spinal muscular atrophy (SMA). Although motor scales can detect disease progression, they may be insufficiently sensitive over short observation periods, particularly in patients with slower progression.

**Objective:**

To assess the prevalence and longitudinal course of fatigue in adult SMA patients using validated instruments. General perceived fatigue was assessed using the Fatigue Severity Scale (FSS), while the Modified Fatigue Impact Scale (MFIS) was used to evaluate multidimensional fatigue—comprising the physical, cognitive, and psychosocial domains. Additionally, we explored associations with motor function, neurophysiological parameters, and treatment status.

**Methods:**

Twenty-five adults with genetically confirmed SMA were evaluated at baseline and after one year using the FSS and MFIS. Motor function was measured by the Hammersmith Functional Motor Scale – Expanded, Revised Upper Limb Module (RULM), and Motor Function Measurement (MFM); neurophysiological assessment included Compound Muscle Action Potential (CMAP), Motor Unit Number Index (MUNIX) and repetitive nerve stimulation. Patients were stratified by fatigue status and use of disease-modifying therapies.

**Results:**

Significant fatigue (FSS > 4) was observed in 60% of patients at baseline and 56% at follow-up. After 1 year, the prevalence of fatigue was significantly lower in treated patients (33.3%) compared to untreated ones (75%; *p* = 0.04). The MFIS scores remained stable across the physical, cognitive, and psychosocial domains. No associations were found between fatigue severity and age, disease duration, motor scale scores, or neurophysiological parameters.

**Conclusion:**

Fatigue is highly prevalent in adults with SMA and does not correlate with disease severity or motor/neurophysiological measures. Patients receiving disease-modifying therapies showed lower fatigue frequency, reinforcing the relevance of fatigue as a meaningful patient-reported outcome in this population.

## INTRODUCTION

Spinal muscular atrophy (SMA) is an autosomal recessive neuromuscular disease caused by biallelic *SMN1* pathogenic variants. The frequency of carriers is estimated at 1/54 in the general population, which leads to a prevalence of 1 per 11,000 for the disease. 1 Proximal lower limb-predominant weakness and hypotonia are the hallmarks of SMA, but age at onset and severity of deficits are variable across the individuals affected. For this reason, SMA is classically divided into 4 subtypes: SMA type 0 includes patients with severe forms, with prenatal onset and survival of less than 1 month; type I patients also have severe forms, starting before 6 months of age, in which patients are unable to sit unsupported; children with type II are able to sit, but never walk unaided; SMA type III presents later, after 18 months of age, and patients reach independent gait. The major determinant of disease severity is the number of copies of a second gene – called *SMN2* : The higher the number, the later the onset. 2 3

Manifestations beyond pure muscle weakness have been lately recognized in SMA. 4 Fatigue, for instance, is now considered a major feature of the disease, particularly in adult patients. 5 Recent data found fatigue to be an almost universal complaint in patients with SMA types II and III. Moreover, it can be so severe as to impact daily activities and overall quality of life. 5 6 7 Despite that, its underlying causes and response to disease-modifying therapies are not clear. 8 In adult patients with SMA, in whom this complaint is even more prevalent, the assessment of fatigue becomes critically relevant. This is especially true for subjects with SMA type III, in whom the disease progression is slow and, therefore, the standard motor scales may fail to capture longitudinal changes, especially in the short term. 9 Nevertheless, these scales are still able to detect progression over longer periods. Hence, detection and quantification of fatigue in this subgroup may be an interesting approach to assess the effects of disease-modifying therapies as a patient-reported outcome. 5 7

Fatigue is a multifactorial phenomenon, involving physical, cognitive, and psychosocial aspects, which can make its objective documentation difficult. 10 11 Specific scales have been developed and validated to quantify fatigue in clinical research settings. The Fatigue Severity Scale (FSS) is a widely used self-administered questionnaire designed to assess the severity of perceived general fatigue and its impact on a person's daily functioning. 10 11 It comprises 9 statements, each rated on a 7-point Likert scale according to which 1 indicates strong disagreement and 7 indicates strong agreement. The final score is the average of the ratings for these items, with higher scores indicating greater fatigue severity. A score above 4 is commonly used as a threshold to signify significant fatigue. The Modified Fatigue Impact Scale (MFIS) is specifically designed to assess separately the impact of fatigue on physical, cognitive, and psychosocial functioning. 12 It consists of 21 items divided into 3 subscales: physical, cognitive, and psychosocial. Each item is rated on a 5-point scale, in which 0 indicates never and 4 indicates that that symptom is almost always present. The total score is the sum of all item ratings, with higher scores indicating a greater impact of fatigue.

Herein, we conducted a longitudinal study to assess the burden of SMA-related fatigue in adult patients using the FSS and MFIS (general perceived fatigue was evaluated through the FSS, and multidimensional fatigue—comprising physical, cognitive, and psychosocial domains—through the MFIS). Furthermore, we explored whether baseline motor function, neurophysiological parameters, and therapeutic status would influence longitudinal change of fatigue severity.

## METHODS

### Selection of subjects

We recruited patients aged 16 years or older with molecular confirmation of SMA and regularly followed up at the Neuromuscular Outpatient Clinic of Universidade Estadual de Campinas (UNICAMP) between 2021 and 2023. Clinical and electrophysiological evaluations of all patients were conducted at baseline and after 1 year by the same physical therapist and the same neurophysiologist. All assessments were performed on the same day for each patient.

The current protocol was approved by the local ethics research committee, and all patients signed an informed consent prior to any study-related procedure.

### Clinical evaluation

Demographic (age/sex) and clinical (age at onset/SMA subtype) data of all enrolled individuals were recorded for further analyses. We also actively searched for all drugs currently in use by each patient, including SMA disease-modifying medications. On the same day, a careful motor and functional evaluation was performed by an experienced physical therapist (CI), who was blind to the results of electrodiagnostic (EDX) tests and treatment status. Motor function was assessed using Hammersmith Functional Motor Scale – Expanded (HFMSE), Revised Upper Limb Module (RULM), and Motor Function Measurement (MFM) 13 14 15 , whereas fatigue was quantified using the Brazilian Portuguese-validated versions of the Fatigue Severity Scale (FSS) and the Modified Fatigue Impact Scale (MFIS). 10 11 Following previous reports, we then stratified patients into 2 groups (with and without fatigue) using a FSS score threshold of 4. This threshold has already been used as a reference in other studies that applied the FSS in neurological disorders. 16

### Electrodiagnostic studies

A board-certified electromyographer (FFG) performed all EDX studies on a NEUROSOFT Neuro-MEP-Micro device (Neurosoft Ltd.). He was blind to all clinical and genetic information at the time tracings were obtained. Motor Unit Number Index (MUNIX) procedures were conducted three times on the Abductor Digiti Minimi (ADM) muscle to assess reproducibility. Initially, the Compound Muscle Action Potential (CMAP) was obtained from the muscle under examination. Subsequently, the patient performed seven levels of progressive isometric muscle contractions (ranging from minimum to maximum) against resistance provided by the examiner, who recorded the electromyography (EMG) surface interference pattern, deriving MUNIX values.

Repetitive low-frequency nerve stimulation of the right ulnar nerve with recording from the abductor digiti minimi muscle was performed in 22 patients, as 3 did not tolerate the procedure.

### Statistical analyses

We used descriptive statistics to report basic demographic, clinical and neurophysiological data of this cohort (median, interquartile range and proportions). Groups with and without fatigue (as defined previously) were then compared regarding age, disease duration, baseline motor scale scores and neurophysiological assessments. Ordinal (non-continuous) variables were assessed using the Wilcoxon Signed-Rank test, and the Fisher's exact test was performed for categorical variables. For all analyses, the significance threshold was set at 0.05. The SYSTAT (Systat Software, Inc.) software, version 13.0, was employed for all statistical analyses.

## RESULTS

Twenty-five adult patients (17 men and 8 women) diagnosed with SMA were included, with 20 patients having type-3 SMA (9 of them being type 3A and 11 being type 3B) and 5 patients having type-2B SMA. The mean age of the patients at baseline was 34.4 years (ranging from 18–57 years), and the mean disease duration was 30.2 years (ranging from 15–52 years). At baseline, 4 patients were undergoing treatment with nusinersen for at least 6 months, whereas after 1 year, 9 patients were undergoing treatment, with 8 on nusinersen and 1 on risdiplam. All newly-treated patients had been on therapy for at least 6 months at the time of follow-up assessment. Nusinersen is administered intrathecally every 4 months after an initial loading phase, while risdiplam is an oral daily medication with systemic distribution 3 ( Table 1 ).

**Table 1:** Demographic and clinical characteristics of the patients at baseline

		SMA (n = 25)
**Age in years: median (range)**		32 (18–54)
**SMA subtype: n (%)**	Type 2	5 (20%)
	Type 3	20 (80%)
**Sex: n (%)**	Male	17(68%)
	Female	8(32%)
**Disease duration in years: median (range)**		28 (15–55)
**Ambulatory: n (%)**	Yes	3 (12%)
	No	22 (88%)
**Treatment at baseline: n (%)**	Nusinersen	4 (16%)
	No treatment	21 (84%)

Abbreviation: SMA, spinal muscular atrophy.

Regarding the prevalence of fatigue, considering a FSS greater than 4, it varied from 60% (15/25) at baseline to 56% (14/25) after 1 year. Comparisons between mean motor scale scores also did not show significant differences over the period, as shown in Table 2 . The mean values of the MFIS subscales are also shown in Table 2 .

**Table 2:** Change in motor and fatigue scale scores between baseline and follow-up

Motor and fatigue scales median (IQR)	Baseline (n = 25)	Follow-up (n = 25)	*p* -value
**MFM-32**	45.83 (30.7–57.3)	44.79 (25.0–59.6)	0.413
**HFMSE**	16 (2.0–32.0)	16 (7.0–31.0)	0.423
**RULM**	23 (13.0–31.0)	29 (9.0–32.0)	0.087
**MFIS physical**	20 (15.0–24.0)	20 (11.0–26.0)	0.423
**MFIS cognitive**	8 (3.0–15.0)	8 (6.0–17.0)	0.193
**MFIS psychosocial**	2 (2.0–4.0)	3 (1.0–4.0)	0.631
**MFIS total**	29 (22.0–41.0)	30 (20.0–40.0)	0.960
**FSS**	4 (3.0–6.1)	5,22 (3.5–6.2)	0.603

Abbreviations: FSS, Fatigue Severity Scale; HFMSE, Hammersmith Functional Motor Scale – Expanded; IQR, interquartile range; MFIS, Modified Fatigue Impact Scale; MFM-32, 32-Item Motor Function Measure; RULM, Revised Upper Limb Module.

Additionally, we evaluated whether clinical and neurophysiological data were associated with fatigue (considering an FSS cutoff value of 4) at baseline. In this comparison, no significant differences were observed between fatigued and non-fatigued patients regarding age ( *p* = 0.824), disease duration ( *p* = 0.781), HFMSE score ( *p* = 1.000), RULM score ( *p* = 0.781), and MFM score ( *p* = 0.803). Likewise, CMAP ( *p* = 0.907) and MUNIX ( *p* = 0.446) values did not show a significant difference between fatigued and non-fatigued individuals. Repetitive nerve stimulation did not reveal any significant decrement in any of the patients evaluated at baseline, which is why it was not repeated during follow-up.

Over one year, fatigue scores (both MFIS and FSS) did not significantly progress in this SMA cohort.

**Figure 1:**
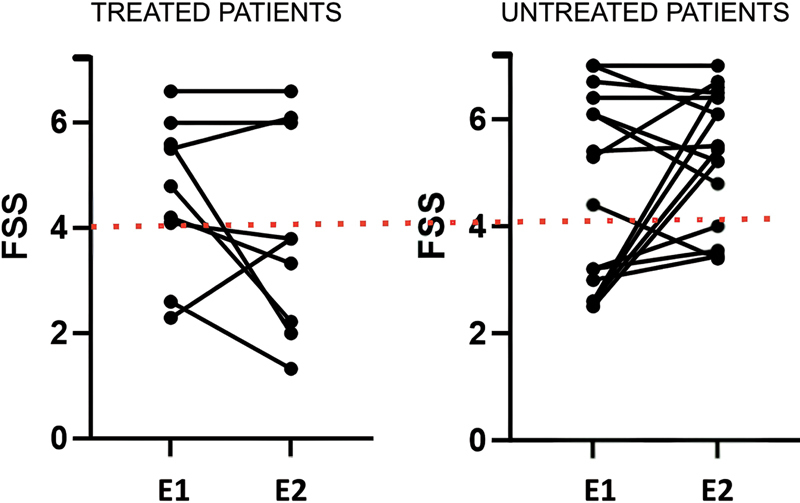
Shows the evolution of the fatigue FSS scale among treated and untreated patients over 1 year and highlights the higher frequency of patients with fatigue (FSS > 4) after 1 year of follow-up in the untreated group. **Abbreviations:** E1, evaluation 1: baseline; E2, evaluation 2: 1 year of follow up).

Finally, we evaluated the impact of treatment by initially comparing the evolution of treated vs untreated groups regarding motor scales, and again, we did not observe significant difference for any of the scales evaluated (HFMSE, RULM, and MFM). For the longitudinal assessment of fatigue in these groups, we compared the number of patients who remained fatigued or became fatigued with those who ceased to be fatigued ( Figure 1 ). In this comparison, it was observed that the proportion of patients with fatigue in the untreated group was significantly higher (75%) compared to the treated group (33.3%), *p* = 0.04.

## DISCUSSION

The present study confirms previous reports on the frequency and clinical relevance of fatigue in adult patients with SMA. 4 7 17 Also, in agreement with the literature, we failed to identify correlations between perceived fatigue and motor function (expressed both by clinical scales and neurophysiological evaluation). This suggests that fatigue and weakness are distinct clinical concepts in this population. In contrast to muscle weakness, fatigue is a subjective construct with multiple contributing factors for each patient. Our results indeed suggest that not only physical, but also psychological issues are relevant. This is shown by the high psychosocial scores of the MFIS scale.

Interestingly, even among patients reporting high fatigue levels, no abnormalities were observed on repetitive nerve stimulation (RNS), which reinforces the multidimensional nature of fatigue. However, this finding does not entirely exclude subtle involvement of the neuromuscular junction, which might only be detectable through more sensitive techniques, such as single-fiber electromyography with jitter analysis. Future studies incorporating these methods may help clarify this potential mechanism.

We also observed that, in adult patients, motor parameters do not significantly change in a year, regardless of treatment status. This finding further supports the concept that disease progression is much slower in this group compared to patients with earlier-onset SMA. So, assessing therapeutic response (or the lack of it) becomes challenging for these patients. Finding novel and more sensitive tools to capture clinical changes—especially in the short term—is clearly needed, and fatigue assessment emerges as an interesting candidate.

Several recent studies have sought to characterize fatigue in SMA patients and evaluate its potential as a relevant outcome measure. Binz et al. 7 (2021) conducted a longitudinal study with adult SMA patients treated with nusinersen, reporting that physical fatigue was the most prominent fatigue dimension, followed by general fatigue and reduced activity. The authors used two instruments: the FSS and the Multidimensional Fatigue Inventory (MFI). A trend toward improvement in some domains was noted after 14 months of treatment, although most fatigue scores remained stable. Importantly, their study lacked a control group of untreated individuals. The presence of a non-treated group in our study may have strengthened our ability to detect treatment-related effects on fatigue and supports the hypothesis that SMN2-targeted therapies may contribute to reducing fatigue burden, at least in part.

Domine et al. (2022), 17 using a novel questionnaire (PROfuture), demonstrated that physical fatigue and perceived fatigability are highly prevalent in adolescent and adult SMA. Their results suggest that both aspects have a significant impact on daily life, particularly in non-sitters. Differently from our study, they found an association between fatigue severity and functional level, likely due to differences in instruments used and cross-sectional study design. Nonetheless, their findings reinforce the multidimensional nature of fatigue in SMA and the importance of considering its various components in patient care and research.

In contrast to the findings by Domine et al. (2022), 17 Binz et al. 7 (2021) observed high fatigue prevalence using FSS and MFI in adult SMA patients but did not find a strong correlation between fatigue and motor impairment, which is consistent with our results. Moreover, some recent studies have explored the use of surface electromyography (sEMG) to objectively assess specifically the motor component of fatigue and its response to specific interventions, offering a promising complementary approach to subjective fatigue measurement. 18 19 20

Taken together, these studies provide a broader context for interpreting our findings and highlight the complexity of fatigue in SMA. The consistency across studies in showing fatigue as a frequent and impactful symptom—often independent of motor status—strengthens the case for incorporating fatigue assessments in clinical trials and routine care.

It is also important to note that we did not include objective assessments of potential confounding factors such as sleep quality, nutritional deficiencies, or depressive symptoms, which may also influence fatigue perception. These dimensions should be systematically explored in future studies to better delineate the multifactorial nature of fatigue in SMA.

Despite limitations due to the small number of patients and the relatively limited sample size in each subgroup, our findings support the clinical impression that treated adult patients experience a reduction in fatigue and, therefore, better quality of life compared to untreated individuals. It is important to acknowledge that, as with any patient-reported outcome, fatigue assessments using FSS and MFIS may be influenced by placebo effects or expectation bias, particularly in unblinded observational studies such as ours. This represents a limitation of our study; however, it is an inherent challenge when using patient-reported outcomes and should be considered when interpreting findings and designing future research.
